# Hazards Analysis, within Departments and Occupations, for Hepatitis B Virus among Health Care Workers in Public Teaching Hospitals in Khartoum State; Sudan

**DOI:** 10.5539/gjhs.v4n6p51

**Published:** 2012-08-26

**Authors:** Taha Ahmed Elmukashfi, Omer Ali Ibrahim, Isam Mohamed Elkhidir, Abdelgadir Ali Bashir, Mohammed Ali Awad Elkarim

**Affiliations:** 1 University of Khartoum, Faculty of Medicine, Department of Community Medicine, Sudan; 2 University of Khartoum, Faculty of Economics, Department of Econometrics and Social Statistics, Sudan; 3 University of Khartoum, Faculty of Medicine, Department of Medical Microbiology and Parasitology, Sudan; 4 Khartoum State Ministry of Health, Sudan; 5 University of Khartoum, Faculty of Medicine, Department of Community Medicine, Sudan

**Keywords:** HBV markers, HCWs, hazardous departments and occupation, public teaching hospitals, Khartoum State, Sudan

## Abstract

**Background:**

Infection with hepatitis B virus (HBV) can lead to a range of clinical illnesses.

**Objectives:**

To examine hazards of hepatitis B virus associated with clinical departments and occupations; among health care workers in Public Teaching Hospitals in Khartoum State, Sudan.

**Methods:**

The study was a cross sectional, facility-based study. It was conducted on stratified two-stage cluster random sample of 843 subjects of whom 324 were at high-hazard, 445 at moderate hazard, and 74 at low hazard; depending on degree of exposure to blood and body fluids of patients. To assess hazards of HBV among departments and occupations of HCWs, non-parametric methods of Chi-square test, was used.

**Results:**

For Anti-HBc vulnerable departments was Renal Dialysis (100%); while for occupations was midwives (73.3%). For carrier rate (+ve HBsAg), highest rate found in department of Management (6.8%); while for occupations was Midwives (6.7%). Regarding immunity (+ve Anti-HBs), the highest percentage found in the department of Dentistry (25.9%); while for occupations was associated with Doctors (14.8%). For a profile of high infectivity (+ve HBeAg), the most vulnerable department in terms of HBV hazards was the Surgery (1.4%); while for occupations was nurses (0.9%).

**Conclusion:**

There was a significant association for infection rate of HBV with occupation and type of department. The most hazardous departments, was Surgery with a profile of high infectivity rate, followed by other departments (medicine, pediatrics, psychiatry & ophthalmology). As for occupations, the most hazardous group was nurses group with a profile of high infectivity rate

## 1. Introduction

There are many causes of hepatitis; examples include alcohol, certain drugs, poisonous mushrooms, and viruses. Hepatitis B was the first hepatitis virus identified by scientists. Infection with the hepatitis B virus (HBV) can lead to a range of clinical illnesses (The Massachusetts Department of public health, 2002; Teo & Lok, 2006; Lok, 2012). Healthcare workers have a high risk of occupational exposure to many blood-borne diseases including HIV, Hepatitis B, and Hepatitis C viral infections. Of these Hepatitis B is not only the most transmissible infection, but also the only one that is preventable by vaccination. HBV infection is a well-recognized occupational risk for an HCW. The risk of HBV infection is primarily related to the degree of contact with blood in the workplace and also to the hepatitis B-e antigen (HBeAg) status of the source person. The risk of HCWs acquiring occupationally related HBV infection has been shown to be associated with several factors. Two important factors are the degree of exposure to the infected body fluids or blood-contaminated sharps such as needles and other medical instruments, and the duration of employment in an occupational risk category (Singhal, Bora, & Singh, 2009). HCW who perform invasive procedures for example surgeons, dentists, emergency workers and those who handle human specimens like the laboratory technicians have been consistently shown to have higher prevalence of hepatitis B virus infection than their counterparts (Ziraba et al., 2010).

According to endemicity of HBV, the world is divided into three areas: Low endemic area (HBsAg prevalence < 2%) in Western Europe and North America; intermediate endemic areas (HBsAg prevalence 2%-7%), in Middle East, Indian Subcontinent; high endemic areas (HBsAg prevalence ≥ 8%), in Sub-Saharan Africa, most of Asia, Pacific, Amazon, and Southern part of Eastern and Central Europe (WHO, N°204, 2008; Alavian et al., 2005; Teo et al., 2006; Lavanchy, 2004).

Serologic markers for HBV were detected in 68% of sexually active heterosexuals in Port Sudan and Suakin (McCarthy et al., 1989). In Khartoum, Sudan, HBsAg was found to be positive in 4% of control-hospitalized patients and 67% in patients with hepatocellular carcinoma (Itoshima et al., 1989). In Khalwat and Salem (two villages, in Gezira State), HBsAg was found in 18.7% and seropositivity for any HB markers (HBsAg, Anti-HBs, or anti-HBc) was found in 63.9% (Hyams et al., 1989). In the South of Sudan (Juba), prevalence of HBsAg was found to be 26% and that of Anti-HBcore is 67% (McCarthy et al., 1994). In a study for Seroprevalence of Hepatitis B and C among health care workers in Omdurman, Sudan; the occupation risk of HBV infection among the HCW in this study was high for the nurses and cleaning staff (Nail, Eltiganni, & Imam, 2008)

HBV is a major infectious occupational hazard of HCWs. HCWs, who considered as carriers, may present a threat to patients (WHO, N°204, 2008; Gully, 1997). In USA, the CDC estimated that in 1985 about 12,000 HCWs were infected with HBV. There is evidence among some groups of HCWs, such as dentists, that rates of exposure are decreasing over time, temporally associated with increased awareness and compliance with the practice of standard precautions (Beltrami, et al., 2000).

A combination of factors believed to be responsible for HBV transmission from HCWs to patients. One factor associated with increased hazard of transmission is the HCW being HBeAg positive, indicating a higher level of infectivity (CPSA Guideline, 1998). Hepatitis B is a well documented occupational hazard for health care workers, including both laboratory and nursing personnel (Sheikh, Hasnain, Majrooh, Tariq, & Maqbool, 2007). Apart from lack of hepatitis B vaccination, nurses and non-professional staff on their own were found to be significantly more susceptible to HBV infection than others (Shrestha & Bhattarai, 2006).

## 2. Objectives

- To measure HBV markers i.e. Anti-HBc, HBs Ag, Anti-HBs & HBe Ag in the blood of HCWs
- To assess hazards of HBV infection among HCWs within the different departments and occupations.

## 3. Materials and Methods

### 3.1 Study Design

It was a cross sectional, facility-based study.

### 3.2 Study Population

Those who joined the work in hospitals for not less than 45 days in 17 Federal Public Teaching Hospitals (6753 HCWs) and 13 State Public Teaching Hospitals (1680 HCWs) in Khartoum State, Sudan. Some of these hospitals have all departments, others have more than one department, and some have only one department.

**The study population was divided into three groups**:

- **High-hazard group:** Those who were working in haemodialysis, surgical departments, blood banks, obstetrical and gynecological departments, dental clinics, laboratories, and ENT departments.
- **Moderate hazard group:** Composed of physicians, pediatricians, and psychiatrists.
- **Low hazard group:** Managerial staff.

This division was based on the degree of contact of the health workers with blood and other body fluids of the patients.

### 3.3 Sampling Frame

The survey or study variable: the prevalence of HBV markers.

Given the objectives of the study and the main variable of interest, two stratification variables were used. These were: (a) type of hospital (Federal or State). (b) Degree of exposure (i.e. type of department).

The degree of exposure was further divided into subgroups: all departments, two departments, one department, in an attempt to increase the internal homogeneity of the groups.

As such, the target population composed of seven strata. Each of the resulting strata contained groups (clusters) of elements belonging (i.e. working) to the departments in hazard groups of different hospitals. This was treated as primary sampling units (PSU).

### 3.4 The Sample Size

Given the study objectives and structure of the population outlined above, the appropriate sample design was a stratified two-stage cluster sampling. Within each of the 7 strata comprising the population, the type of department of different hospitals in the stratum form the PSU from which hospitals was selected at random with probabilities proportionate to their sizes (PPS) (i.e. number of workers). A number of HCWs, then, was selected from the PSU.

The formula for determining the overall sample size in this case is:


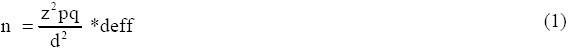


z=confidence coefficient =2.

p= prevalence rate= 50%.

q = (1 –p) = 50%,

d (desired margin of error) = 0.05.

deff =the design effect =2 for stratified two-stage design.

Accordingly: 
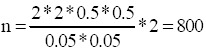


So, to estimate the prevalence rate with a 95% confidence and with a margin of error, that does not exceed 0.05, we need a sample of 800 HCWs.

The overall sample size allocated proportionately to the strata comprising the population.

### 3.5 Sample Selection

It was conducted on stratified two-stage cluster random sample.

**First**

The hospitals were divided according to the type of the hospitals into two groups:

- Federal Teaching Hospitals with a total number of 6753 HCWs.
- State Teaching Hospitals with a total number of 1680 HCWs.

**Second**

The hospitals were divided into groups according to the different departments in each.

The ratio of HCWs in the Federal Hospitals to those in the State Hospitals was 4: 1. Therefore, the sample size taken from the Federal Hospitals was 640 HCWs and that from the State Hospitals was 160 HCWs.

The number of selected hospitals was as follows:

- Three, one, two, and four hospitals were selected from each group of Federal Public Teaching Hospitals respectively by PPS. Giving a total of ten hospitals.
- Two, two, and three hospitals were selected from each group of State Public teaching Hospitals respectively by PPS. Giving a total of seven hospitals.

Each hospital was divided into strata depending on the different departments in it. Then the sample size was divided among these strata according to the HCWs size in each. HCWs from each department were selected by the simple random method.

The calculated sample size (800) was divided proportionately among the selected hospitals according to the size of the HCWs. This sample size was calculated after approximation of the fraction for each hospital, and was found to be equal to (600+208=808). This sample was further divided among the different departments of each hospital with approximation of the fraction for one decimal point. So the final sample size (actual sample size) was (639+231) = 870 HCWs.

### 3.6 Tools of Data Collection

#### 3.6.1 Informed consent from the selected HCWs was obtained.

#### 3.6.2 Survey

A pre structured questionnaire for the HCWs in Public Teaching Hospitals in Khartoum State. It included the demographic profile characteristics; departments and the type of the medical department.

#### 3.6.3 Laboratory Blood Tests

To measure HBV markers i.e. Anti-HBc, HBsAg, Anti-HBs & HBeAg in the blood of HCWs in Public Teaching Hospitals in Khartoum State, Five mills of venous blood was collected using 10 ml vaco container. Sera was separated and stored at –20° centigrade, until testing. ELISA was used to screen for anti HB core total. Reactive specimens for anti HB core were tested for HBs Ag. Reactive specimen for HBs Ag was tested for HBeAg. Vaccinated HCWs and part of the reactive specimens for anti- HB core but non-reactive for HBs Ag was tested for Anti-HBs.

### 3.7 Statistical Approach

Data was entered and analysed using SPSS program. P≤0.05 was considered statistically significant. The study used non-parametric methods of Chi-square test to validate the results.

## 4. Results

A total sample of 870 was collected; 27 specimens were lost. Therefore, the final sample size of the study was 843 HCWs. The response rate was 96.9%. Out of them 628 HCWs (74.5%) from Federal Public Teaching Hospitals, while 215 HCWs (25.5%) from Khartoum State Public Teaching Hospitals.

The age group representation was 58.4% for the age group 30-49, followed by 30.7% for less than 30 years, and the least one (10.9%) was for the age group of 50+ years. The gender representation was 366 males HCWs (43.4%) and 477 females HCWs (56.6%). Regarding education; 269 HCWs (31.9%) had university education, followed by high secondary education with 214 HCWs (25.4%) while only 5 HCWs (0.6%.) had Quranic School (khalwa) education. 460 HCWs (54.6%) were married, 381 HCWs (45.2%) were unmarried and 2 HCWs (0.2%) refused to identify themselves. With regard to vaccination, 92% of them were not vaccinated while only 8% were vaccinated.

Regarding occupation, the study population composed of 322 (38.2%) nurses, 209 (24.8%) doctors, 15 (1.8%) midwives, 42 (5%) Lab. + Blood bank employees and 72 (8.5%) laborers. The distribution of the study population among the different departments was found to be as follow: 138 (16.4%) Surgery, 106 (12.6%) Obstetric & Gyneocology, 27 (3.2%) Dentistry, 5 (5.9%) Pathologist, 74 (8.8%) Management, 3 (0.4%) Renal dialysis, and 445 (52.8%) Other departments (medicine, pediatrics, ophthalmology and psychiatry), Management department represents all HCWs that were not in direct contact with patients.

**Hazard analysis within departments and occupations:**

We analyzed the hazard factors across departments and occupations, so that we identified the most vulnerable hazard groups, in terms of department and occupation, using Chi-square test.

**Figure 1:**
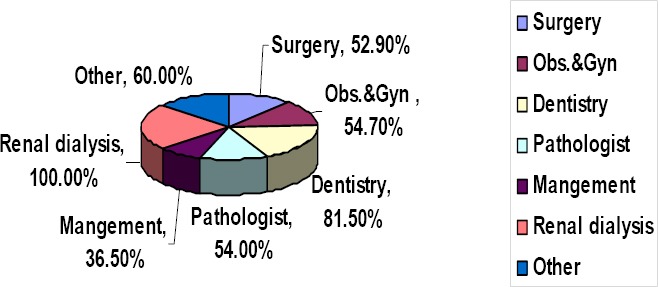
Illustrates distribution of Anti-HBc within departments (N = 843)

From Table 1, regarding departments, the highest percentage of positive Anti-HBc was associated with Renal Dialysis (100%) followed by Dentistry (81.5%), Other departments (60.0%), Obstetric & Gynecology (54.7%), and Surgery 52.9%. The least percent was from the management (36.5%), see figure (1). For occupations, the highest is midwives (73.3%), followed by laborers (67.8%), theatre attendance (64.7%), doctors (58.4%), and nurse (54.0%).

**Table 1:** Distribution of Anti-HBc across departments and occupations of HCWs

Department	HCWs occupation							

	Doctor	Nurse	Midwives	Lab.+blood Bank employers	Labor	Theatre attendants	Managers	Total
Surgery	35.6	37.0	0.0	0.0	6.8	20.5	0.0	52.9
Obs.&Gyn	24.1	19.0	19.0	0.0	29.3	8.6	0.0	54.7
Dentistry	54.5	18.2	0.0	0.0	18.2	9.1	0.0	81.5
Pathologist	0.0	11.1	0	81.5	7.4	0	0.0	54.0
Management	0	0	0	0	7.4	0	92.6	36.5
Renal dialysis	0	0	0	0	100.	0	0.0	100.0
Other departments	26.2	48.3	0	0	25.5	0	0.0	60.0
Total	58.4	54.0	73.3	52.4	67.8	64.7	34.7	

* Results are significant at 5% according to Chi-square test

With regard to occupation, it was obvious that the highest percent of Anti-HBcore was found with Midwives (73.3%), followed by laborers (67.8%), and Theatre Attendants (64.7%). The least percent was associated with the managers (34.7%).

Regarding HBsAg Table 2 showed that according to departments the highest carrier state was associated with the department of Management (6.8%), followed by other departments (3.4%) and surgery (2.9%). On the other hand, for occupations the highest carrier percentage was associated with Midwives (6.7%), followed by Managers (5.6%), and Nurses (4.7%).

**Table 2:** Distribution of HBsAg across departments and occupations of HCWs

Department	HCWs occupation							

	Doctor	Nurse	Midwife	Lab.+ blood Bank employers	Labourers	Theatre attendants	Managers	Total
Surgery	25.0	75.0						2.9
Obs.&Gyn		66.7	33.3					2.8
Dentistry								0.0
Pathologist								0.0
Management					20.0		80.0	6.8
Renal dialysis								0.0
Other departments	6.7	66.7						3.4
Total	1.0	4.7	6.7	0.0	3.4	0.0	5.6	

*Results are significant at 5% according to Chi-square test

Table 3 reflected the immunity with regard to departments and occupations. The highest percentage of +ve Anti-HBs had been found in the department of Dentistry 25.9%, followed by Surgery 9.4%, and Obstetric & Gynecology 7.5%. Concerning occupations the highest percentage of +ve Anti-HBs was associated with Doctors 14.8%, followed by Midwives 6.7%, and theatre attendants 5.9%.

**Table 3:** Distribution of Anti-HBs across departments and occupations of HCWs

Department	HCWs occupation							

	Doctor	Nurse	Midwife	Lab.+ blood Bank employers	Labour	Theatre attendants	Managers	Total
Surgery	76.9	15.4				7.7		9.4
Obs.&Gyn.	50.0	12.5	12.5		12.5	12.5		7.5
Dentistry	100.0							25.9
Pathologist				100.0				2.0
Management								1.4
Renal dialysis								0.0
Other	62.5	37.5						3.6
Total	14.8	2.8	6.7	2.4	0.7	5.9	1.4	

*Results are significant at 5% according to Chi-square test

Table 4 indicated that, the most hazardous department was Surgery, with a profile of high infectivity rate of 1.4%, followed by other departments (medicine, pediatrics, ophthalmology and psychiatry) which scored 0.2%. Regarding occupations, the most hazardous group was nurses with a profile of high infectivity rate of 0.9%.

**Table 4:** Distribution of HBeAg across departments and occupations of HCWs

Department	HCWs occupation							

	Doctor	Nurse	Midwife	Lab. and blood Bank employers	Labour	Theatre attendants	Managers	Total
Surgery		100.0						1.4
Obs.&Gyn								0.0
Dentistry								0.0
Pathologist								0.0
Management								0.0
Renal dialysis								0.0
Others		100.0						0.2
Total		0.9						

*Results are significant at 5% according to Chi-square test

## 5. Discussion

### 5.1 Infection Rate

There was a significant association for infection rate of HBV with occupation and type of department.

Among the HCW’s occupations, midwives registered the highest percentage of positive Anti-HBc prevalence, followed by laborers and theatre attendants. The minimum prevalence of infection was found among managers. The more HCWs subjected to hazards of contact with blood and body fluids of patients the more was the chance for them to be infected. It was similar to previous studies as revised by (Teo et al., 2006)

With regard to department, the highest rate of infection was associated with renal dialysis, followed by Dentistry, Obstetric & Gynecology and Surgery, while the management occupies the tail end of the prevalence. This supported the fact that the department mostly involved in contact with patient’s body fluids and blood, the more was the chance for HCWs to get HBV infection. Similar study showed the same result as published by (Teo et al., 2006).

### 5.2 Prevalence of HBsAg

Occupation and type of department were found to be important hazard factors for carrier state. Similar studies at international level got similar findings as recorded by (WHO, 2008); and by a study done in Canada (Gully, 1997)

The highest carrier rate was recorded among the managers of the hospitals, followed by midwives and nurses. Although the operation theatre attendants were reported number 3 in the infection rate, they got the lowest carrier rate together with Laboratories and blood bank employees. Regarding managers, it may be due to a balanced increased hazard of occupational exposure, because HBV was endemic in Sudan. While for midwives and nurses, it may be due to their direct contact with blood and body fluids of the patients and with minimal measures of precautions. The reason, also, might be that safe life styles and appropriate job behavior of doctors, due to their higher level of education and socioeconomic factors as compared to the rest of HCWs in such communities.

As far as type of department was concerned, it was found that the department of management scored the highest carrier rate, followed by other departments (medicine, pediatrics, ophthalmology and psychiatry) and Surgery; while Dentistry, Renal and Pathologist had the least carrier rate. The high carrier rate of HBV in other departments may be these HCWs practiced, at one time, shift of work among departments where contact with blood and body fluids of patients was more; or may be due to low safety measures in their departments.

### 5.3 Immunity Rate

For the effect of departments and occupations on the rate of positive Anti-HBs, the highest rate was found in the department of Dentistry, followed by Surgery, and Obstetrics & Gynecology. As for occupations, the highest rate of immunity was found to be associated with Doctors, followed by Midwives and Operation Theatre Attendants.

These were departments, where HCWs were more exposed to contact with blood and body fluids of patients. Therefore, the high immunity rate, here, may indicate vaccination and / or post infection immunity.

### 5.4 Profile of High Infectivity Rate

Regarding analysis within departments and occupations the most hazardous groups in terms of department and occupation, using chi-square test, was Surgery with a profile of high infectivity rate, followed by other departments (medicine, pediatrics, ophthalmology and psychiatry). As for occupations, it was found that the most hazardous group with a profile of high infectivity rate was the nurses group. Nurses are closer to patients on a routine basis. This finding is in line with other studies (Ziraba et al., 2010; Nail et al., 2008).

## 6. Conclusion

The most hazardous department for infection rate was renal dialysis, for carrier rate was management, and for a profile of high infectivity rate was Surgery; while the most hazardous occupation for infection rate was paramedical staff, for carrier rate was midwives and for a profile of high infectivity rate was nurses group. So, the most hazard groups in terms of departments, was Surgery with a profile of high infectivity rate, while for occupations, the most hazardous group was nurses group.

The main implication of this study is that vaccination of health care workers against HBV is required before and during training in schools of medicine, dentistry, nursing, laboratory technology, and other allied health professions, and before trainees have their first contact with patient’s blood and body fluids. Also there is an imperative need to establish an infection control unit in each hospital to be responsible for implementing and monitoring infection control system in each hospital.
